# Mutual modulation between surface chemistry and bulk microstructure within secondary particles of nickel-rich layered oxides

**DOI:** 10.1038/s41467-020-18278-y

**Published:** 2020-09-07

**Authors:** Shaofeng Li, Zhisen Jiang, Jiaxiu Han, Zhengrui Xu, Chenxu Wang, Hai Huang, Chang Yu, Sang-Jun Lee, Piero Pianetta, Hendrik Ohldag, Jieshan Qiu, Jun-Sik Lee, Feng Lin, Kejie Zhao, Yijin Liu

**Affiliations:** 1 Stanford Synchrotron Radiation Lightsource, SLAC National Accelerator Laboratory, Menlo Park, CA 94025 USA; 2 State Key Lab of Fine Chemicals, School of Chemical Engineering, Liaoning Key Lab for Energy Materials and Chemical Engineering, Dalian University of Technology, 116024 Dalian, China; 3 School of Mechanical Engineering, Purdue University, West Lafayette, IN 47907 USA; 4 Department of Chemistry, Virginia Tech, Blacksburg, VA 24061 USA; 5 Department of Geological Sciences, Stanford University, Stanford, CA 94305 USA; 6 Advanced Light Source, Lawrence Berkeley National Laboratory, Berkeley, CA 94720 USA; 7 Department of Material Sciences and Engineering, Stanford University, Stanford, CA 94305 USA; 8 Department of Physics, University of California-Santa Cruz, Santa Cruz, CA 95064 USA

**Keywords:** Batteries, Nanoscience and technology

## Abstract

Surface lattice reconstruction is commonly observed in nickel-rich layered oxide battery cathode materials, causing unsatisfactory high-voltage cycling performance. However, the interplay of the surface chemistry and the bulk microstructure remains largely unexplored due to the intrinsic structural complexity and the lack of integrated diagnostic tools for a thorough investigation at complementary length scales. Herein, by combining nano-resolution X-ray probes in both soft and hard X-ray regimes, we demonstrate correlative surface chemical mapping and bulk microstructure imaging over a single charged LiNi_0.8_Mn_0.1_Co_0.1_O_2_ (NMC811) secondary particle. We reveal that the sub-particle regions with more micro cracks are associated with more severe surface degradation. A mechanism of mutual modulation between the surface chemistry and the bulk microstructure is formulated based on our experimental observations and finite element modeling. Such a surface-to-bulk reaction coupling effect is fundamentally important for the design of the next generation battery cathode materials.

## Abstract (web summary)

The interplay of surface chemistry and bulk microstructure in layered oxides is critical to battery performance. Here, the authors demonstrate a comprehensive understanding of such a reaction mechanism within an individual cathode particle using integrated synchrotron imaging methods.

## Introduction

Lithium ion batteries (LIBs) are groundbreaking development in the energy storage technology that has substantially impacted the modern society. The applications of LIBs in consumer electronics and electric vehicles have motivated tremendous research efforts in this field. Among all the components of the battery, the cathode remains the most significant limiting factor for further improvements in the energy and power densities, two of the most important performance attributes concerning electric vehicle applications^1^. Comparing to other cathode candidates for the next-generation battery, Ni-rich NMC (LiNi_x_Mn_y_Co_z_O_2_; *x* + *y* + *z* ≈ 1, *x* ≥ *y* + *z*) layered oxides exhibit advantages in their practical energy densities (in some cases it could reach beyond 200 mAh g^−1^)^2^. The reduction in the amount of cobalt used in the Ni-rich cathode is also important not only from a cost-efficiency perspective but also due to the dubious ethics of cobalt mining^3^. Although the incentives to use Ni-rich NMC are significant, these materials suffer from unsatisfactory capacity retention upon prolonged cycling with high-voltage charging. The lithium intercalation/deintercalation could lead to unwanted phase transformations that degrade the performance, especially at high delithiation state. These undesired side reactions are more severe in the Ni-rich compound than in the widely studied and commercialized LiNi_1/3_Mn_1/3_Co_1/3_O_2_^4^.

It has been reported that the surface chemistry plays a vital role during battery operation, although the battery operation is ultimately a bulk chemical process (i.e., the lithium ions (de)intercalate into the bulk of the active material). The undesired surface reactions include, but are not limited to the, reconstruction of the surface lattice structure^5–8^, the formation of a reaction passive interface^9^, dissolution and precipitation of metal cations^10^, growth of lithium dendrites from the particle surface etc^11^. These surface chemical processes lead to the development of local impedance and effectively cause the lithium ions and the electrons to detour through geometrically less optimal pathways, result in unwanted phenomena like cell polarization and capacity/voltage fade^12^. Through such mechanisms, they affect the participation level of the active particles in the cell scale chemistry. While the importance of surface chemistry is well appreciated, a systematic study of the heterogeneous reaction pattern over the surface of individual particles is not yet available. More importantly, how does the bulk microstructure interact with the surface chemistry is an outstanding question yet to be addressed. Such a study is nontrivial because it requires a suite of complementary and advanced experimental tools with varying probing depth, nanoscale spatial resolution, and sufficient chemical sensitivity. These requirements urge further developments in the microscopic technique that integrate the advantages of existing imaging modalities, which is a frontier research trend that has attracted immense research interest in recent years^13–16^. It is worth mentioning that the operando high-resolution X-ray diffraction-computed tomography (XRDCT) has been demonstrated as a promising approach to quantify crystallographic heterogeneities spatially and temporally within and between the electrode particles^17^. We would like to highlight that, although the size of the X-ray focal spot (i.e. the nominal spatial resolution) for the XRDCT technique may not be very fine, this technique is sensitive to the material’s lattice structure, which is directly related with the atomic-scale structural and chemical evolution of the cathode material upon electrochemical cycling.

Herein, we tackle this question by a systematic investigation of a single LiNi_0.8_Mn_0.1_Co_0.1_O_2_ (NMC811) secondary particle at its charged state using multiple X-ray probes with nanoscale spatial resolution in both soft and hard X-ray regimes. Full-field transmission hard X-ray microscopy (TXM) was used to reconstruct the three-dimensional (3D) microstructure of the particle with nominal spatial resolution down to ~30 nm^18^. A scanning soft X-ray nanoprobe was used to map out the surface Ni valence state of the same particle with probing depth of ~5 nm and lateral spatial resolution of ~30 nm^19^. Both measurements were coupled with the energy tunability of the synchrotron source, offering valuable insights into the local chemistry through extracting the local spectroscopic fingerprints. Our results offer a direct visualization of the structural and chemical complexity throughout a single NMC811 secondary particle. It appears that the degree of surface lattice reconstruction (from the layered structure to a mixture of spinel and rock-salt structures) is inhomogeneous over the particle surface and, more interestingly, is correlated with the bulk porosity over the corresponding regions. Our finite element modeling (FEM) further shed some light on the mechanism of the mutual modulation between the surface chemistry and the bulk microstructure. Such a surface-to-bulk correlation highlights the importance of the particle’s mechanical robustness because the formation of morphological defects (the micro cracks) within the particle not only modulates the internal charge and strain distribution, but also interacts with the local surface chemistry. Our work, first of its kind, suggests that both crack mitigation and surface modification are key points that shall be addressed in a comprehensive and integrated strategy for the design of next-generation battery cathode materials.

## Results

### Nano-probing the local chemistry in a charged NMC811 particle

**Fig. 1:**
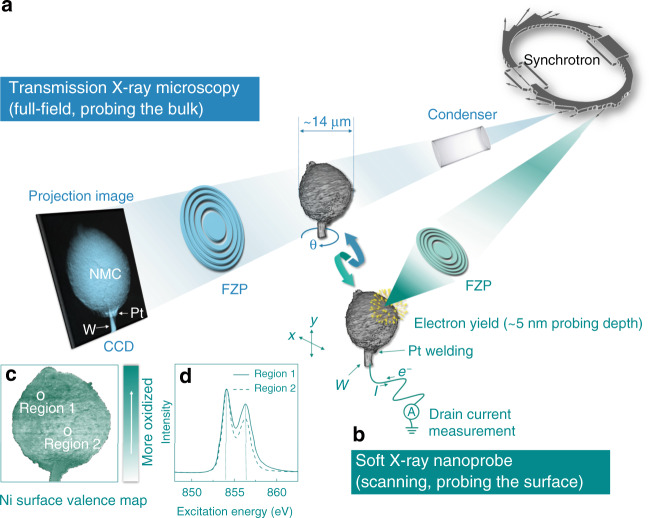
Schematic illustration of the correlative imaging for an NMC811 secondary particle. In the full-field TXM measurement (**a**, light blue color), the transmission images of the particle were acquired as the sample is rotated in a tomographic scan. In the soft X-ray nanoprobe measurement (**b**, dark green color), the total electron yield (TEY) signal is recorded by measuring the drain current. Through raster scanning the particle with respect to the focal point, the surface chemical inhomogeneity (**c**) is revealed based on the energy-dependent local TEY signal (**d**) over different surface regions.

As discussed above, the local chemistry within individual cathode particle is complicated yet impactful. The fundamental question regarding the sub-particle level structural and chemical interplay urges for nano-probing the particle with multi-modal signal detection in order to achieve sufficient sensitivities to different aspects of the particle properties. Synchrotron-based nano- resolution X-ray imaging methods have been well-established as a suite of powerful tools for studying the battery particles. In particular, the full-field TXM (Fig. 1a), which utilizes a Fresnel zone plate as the objective lens to achieve full-field imaging at spatial resolution down to ~30 nm, has found broad applications in the mesoscale battery science^20^. By coupling the full-field imaging with the X-ray energy scan, two-dimensional (2D) or 3D spatially resolved spectroscopic signals can be extracted, relating the local valence state of the elements of interest (often used as a proxy of the local state of charge (SoC)) to the local morphological features^21^.

With the detection of the transmission signal, the TXM technique non-invasively reconstruct the interior of the particle, revealing the bulk characteristics of the particle. In addition, it is possible to explore surface chemistry by employing soft X-ray nanoprobe (Fig. 1b). The utilization of the TEY signal in soft X-ray nanoprobe measurement further reduces the probing depth to only ~5 nm and significantly promotes the sensitivity of this technique to the local surface reactions. Illustration of nano-resolution surface chemistry mapping can be found in Fig. 1c and Supplementary Fig. 1, in which the energy-dependent TEY signal (Fig. 1d) is used to fingerprint the local surface Ni valence state, resulting in chemical contrast between the labeled region 1 and region 2. It is clear that an integrated and correlated soft and hard X-ray imaging approaches can offer a unique opportunity for a comprehensive understanding of the battery cathode particle’s behavior. Therefore, we implement such an unprecedented experimental combination to elucidate the reaction coupling effect between the surface chemistry and the bulk microstructure within a unique individual NMC811 secondary particle (see illustration in Fig. 1). More systematic discussion about such surface-to-bulk reaction coupling effect will be presented below.

### The surface chemical degradation in the NMC811 electrode

It was well-known that side reactions of the NMC811 cathode with the liquid electrolyte, could cause the rearrangement of the layered lattice structure, forming kinetically unfavorable spinel and/or rock-salt phases and resulting in a reduced valence state at Ni^2+^ ^22,23^. Such a valence state change has a distinct contribution to the spectroscopic fingerprint in the soft X-ray absorption spectroscopy (XAS) data over the Ni *L*_3_-edge. It is worth noting that, without lateral spatial resolution, exploring the chemical reaction at different depths has been demonstrated using the conventional XAS signals in two different detection modalities, e.g. TEY (~5 nm probing depth) and FY (fluorescence yield, ~100 nm probing depth)^3^. In our NMC811 case, the peak at excitation energy of ~854.0 eV is enhanced in the TEY spectra compared to the FY spectra (Supplementary Fig. 2a, b). It indicates that a relatively higher portion of Ni^2+^ is distributed on the particle surface. Moreover, comparison between the samples recovered after the 1st and the 46th cycles reveals that the repeated electrochemical cycling could exacerbate chemical degradation over the particle surface. These Ni spectroscopic signals are also cross checked using the XAS spectra measured over the oxygen *K*-edge. It has been reported that the NMC lattice reconstruction is accompanied by the suppression of the pre-edge peak at excitation energy of ~530.3 eV^3^, which is consistent with our spectroscopic results (Supplementary Fig. 2c, d). Note that we are actually monitoring the Ni valence behavior in O *K*-edge XAS through the Ni 3*d*-O 2*p* hybridization. In our observation, Ni^3+^-O^2−^ hybridization state is weaker after prolonged cycling (the peak at excitation energy of ~530.3 eV, black arrows in Supplementary Fig. 2c, d), indicating a suppressed Ni^3+^ state, which is consistent with the implication of the Ni *L*_3_-edge spectra.

### The microstructure and charge distribution in the NMC811 particle

While the surface chemistry can be studied using soft X-ray spectroscopy, investigation of the bulk formation of the NMC811 particles requires hard X-ray tools due to the required penetration capability. The NMC811 secondary particles are agglomerations of fine grains formed in a self-assembly process during the synthesis. They are, therefore, populated with grain boundaries^16^, intergranular and intragranular cracks^12^, charge heterogeneity^23^, and inhomogeneous mechanical strain fields^24^. The anisotropic lattice breathing during the repeated electrochemical cycling could lead to a buildup of strains and lattice defects that are eventually released through particle disintegration^25^. The morphological and chemical defects could, in turn, affect the diffusion kinetics^26,27^, resulting in a degradation in electrochemical performance. We employed the TXM to probe the NMC811 particle’s internal microstructure and charge distribution in a non-invasive manner.

**Fig. 2:**
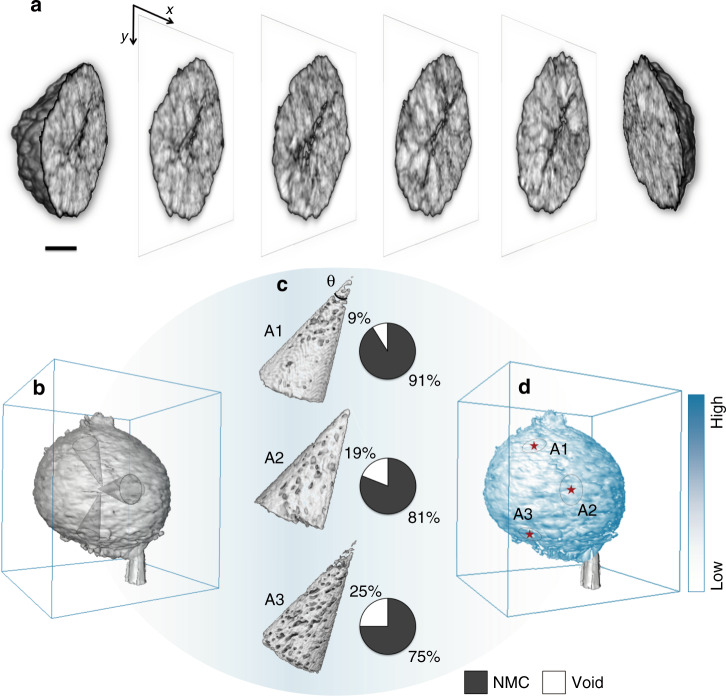
Structural complexity within a charged NMC811 secondary particle. **a** 3D rendering of the tomographic data over the particle with the perspective views of a few virtual slices through different depths displayed in the center. **b** The three cone-shape cutout regions from the nano-tomography data. **c** Schematic of the local porosity, which is defined by the volume ratio of the void within the respective cone regions. **d** The calculated porosity map, which shows the porosity value assigned to the surface of particle. The angle θ can be tuned to balance the signal-to-noise and the lateral resolution. The presented maps are based on the calculation with θ set to be 3^o^. The scale bar in **a** is 5 μm.

As illustrated in Fig. 2a, the cracks are ubiquitous in this particle. For a better evaluation of the correlation between the internal microstructure and the bulk charge distribution, the morphology and the Ni K-edge energy map over the virtual xy-slice through the center of the particle are shown in Supplementary Fig. 3a, b, respectively. While highlighting the mesoscale complexity, a depth-dependent Ni oxidation state distribution is clearly observed in Supplementary Fig. 3b, and is quantified in Supplementary Fig. 3c (the averaged depth profile) and Supplementary Fig. 3d, e (selected line profiles from the surface to the center). It is clearly observed that the near-surface regions are generally more oxidized comparing to the particle core. Such a depth profile suggests that majority of the charge transfer between this particle and the external environment happens at the outer surface. More importantly, with the input from the nano-tomography data, we extract the cone-shape regions (with the apex located at the center of the particle and the opening angle at 3°) and further calculate the porosity (the volume ratio of the pore in each cone region) within the cut-out regions (Fig. 2b–d). The calculated porosity value is then assigned to the center of the cone base, which is located on the particle surface. This procedure is repeated for all the surface pixels and the color-coded porosity map is presented in Fig. 2d.

### The correlation between local surface chemistry and bulk microstructure

**Fig. 3:**
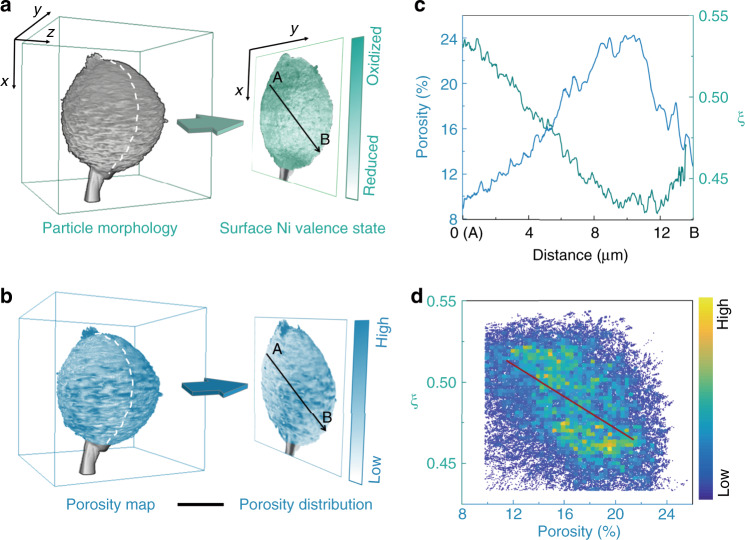
The correlation between the local surface reconstruction and the bulk porosity. **a** The particle morphology and the surface (~5 nm depth) Ni valence state distribution. **b** The bulk porosity map. **c** The line profile from points A to B as illustrated in **a**, **b**. The *ξ* is the ratio between the TEY intensity at 856.2 eV and the sum of that at 854.0 eV and 856.2 eV pixel by pixel, which represents relative Ni valence state. **d** The correlation plot for all the data points in **a**, **b**. The plot is color coded to the density of the data point (see colormap in the inset). A weak but clear negative correlation (see regression line in **d**) is observed between the surface Ni valence state and the bulk porosity. The scale bar in **b** is 5 μm.

For a fundamental understanding of the interplay between the local surface chemistry and the bulk microstructure, we demonstrate a correlative nano-resolution imaging approach in both soft and hard X-ray regimes. It is essential to execute such correlative imaging approach at single-particle scale, because the statistically averaged information, e.g. the soft/hard XAS spectra at the electrode scale, could miss some key points for decoupling the delicate correlation between the local surface chemistry and the bulk microstructure. The NMC811 particle shown in Fig. 2 was also imaged using the scanning soft X-ray nanoprobe (Fig. 3a). We tuned the incident X-rays to two excitation energies at 854.0 eV and 856.2 eV and collected the corresponding TEY maps (Supplementary Fig. 1a–c). X-rays at these two excitation energies have subtle difference in the TEY response. The relative TEY intensities at these two energies fingerprint the valence state of the local Ni species (Supplementary Fig. 1d, e).

To quantify the contrast in the local Ni valence state, we calculate the ratio (*ξ*) between the TEY intensity at 856.2 eV and the sum of that at 854.0 and 856.2 eV pixel by pixel and used it to color code Fig. 3a (calculation details can be found in Supplementary Fig. 1). We clearly observed a position-dependent variation of Ni valence state (the *ξ* maps of Supplementary Fig. 1d and the valence state maps of Supplementary Fig. 1e), suggesting an inhomogeneous lattice reconstruction effect over the surface of the scanned particle. We point out that, in practice, we conducted the soft X-ray nanoprobe (Fig. 3) prior to the hard X-ray nano-tomography (Fig. 2) on the same particle. This is because the surface chemical mapping using soft X-ray nanoprobe is more sensitive to the sample environment (e.g. potential air exposure).

Such correlated measurements over this unique particle allow us to do a one-to-one correlation between the surface valence mapping (Fig. 3a) and the bulk porosity mapping data (Fig. 3b). The line from A to B denotes a single-pixel-wide line in the projected 2D images. Over this line, we plot the intensity profiles based on the surface valence mapping and the bulk porosity mapping data, respectively. The pixel sizes of the data from the two imaging methods are very close but not identical. We utilized the bilinear interpolation method to rescale the matrix slightly for a pixel-to-pixel matching. Direct visual assessment of Fig. 3a, b suggests that there is a negative correlation between the surface Ni oxidation state and the porosity of the corresponding cone region. For a better evaluation of this correlation, the line profiles from points A to B was first plotted in Fig. 3c, which clearly indicates the inverse trends in the position-dependent profiles. For better statistics, the correlation plot for all the pixels over the surface of this particle is shown in Fig. 3d. The data points are quite broadly scattered, suggesting a relatively weak correlation, but the negative trend is unambiguous (see the red line in Fig. 3d for the linear regression fitting; the Pearson correlation coefficient is quantified to be −0.509).

It should be noted that the Li-ion’s diffusion pathways within a secondary NMC particle is rather complicated. This is because the randomly oriented primary grains in the secondary particle not only define the intra-grain optimal diffusion directions for Li-ions, but also induce ubiquitous grain boundaries and structural complexities that further complicate the heterogeneous and anisotropic energy barriers for the inter-grain Li-ion diffusion. The lattice defects including the local short-range ordering can also affect the local resistance for the Li diffusion^27^. In addition to the particles’ intrinsic structural complexities, the in situ formed particle cracks can cause liquid electrolyte infiltration, forming new solid-electrolyte interphase that could facilitate the Li-ion transportation in the short term but build-up impedance over the long run^12^. When projecting the bulk porosity to the particle surface using the methodology described in Fig. 2, it is important to take the above-discussed complexity into consideration and choose the cone volume with properly selected shape. Therefore, in our approach, we adjust the size of the bottom of the cone by changing the opening angle (0.6–6^o^) to reflect the degree of the tortuosity in the Li-ion’s outward diffusion pathways. When the opening angle is set to relatively small, it indicates a more radical and less torturous outward Li diffusion. As shown in Supplementary Fig. 4, an optimal opening angle of 3^o^ is selected by evaluating the degree of correlation between the directly measured soft X-ray nanoprobe surface mapping data and the numerically projected bulk porosity onto the particle surface. We acknowledge that engineering of the NMC secondary particles with purposely aligned primary grains could impact our results and our model needs to be adjusted accordingly in those cases^28^.

We would like to point out that there could be redox heterogeneity at a length scale finer than our spatial resolution (~30 nm). Due to the limited spatial resolution and the nature of our techniques, we are not able to reveal the sub-pixel level chemical heterogeneity. In our soft X-ray nanoprobe data, a surface pixel is represented by one number, which is the averaged valence state over the corresponding surface pixel. There are some imaging methods that could potentially offer sub-pixel level information. For example, in the XRDCT technique^17^, after measuring the local XRD pattern associated with a certain pixel, it is possible to refine the XRD data to evaluate the broadening of the diffraction peaks, which correlates with the sub-pixel lattice disordering. The X-ray tensor tomography is another method that could offer sub-pixel level information by evaluating the critical feature size and orientation from the small angle scattering pattern^29^. These imaging methods can also offer valuable information for the study of battery materials.

### Theoretical insight into the surface-to-bulk mutual modulation

To understand the mutual modulation between charge heterogeneity, bulk fracture, and surface passivation at the single-particle level, we compute the co-evolution of the Li/charge distribution, stress, and intergranular fracture in an NMC particle upon charging using finite element analysis. The detailed procedure is described in the Methods section. Briefly, Li concentration is determined by the kinetics of diffusion. The mechanics field is solved by the standard equations of deformation kinematics, constitutive law, and the momentum equilibrium. The progressive evolution of the intergranular fracture is represented by the damage function along the grain boundaries. The NMC secondary particle is constructed as an aggregation of single-crystalline primary particles of random sizes and grain orientations. Li diffusion and the Li insertion/extraction induced lattice change in NMC is highly anisotropic. Furthermore, the mechanical property of single-crystalline NMC is intrinsically anisotropic. These anisotropic aspects of Li transport, mechanical deformation, and elastic parameters are critical to capture the feature of charge distribution and mechanical failure in the NMC secondary particle. The model parameters used in the FEM are summarized in the Supplementary Table 1.

We demonstrate the positive feedback between charge heterogeneity and intergranular fracture in an NMC secondary particle. Four different constant-concentration boundary conditions in each quarter domain of the particle surface are prescribed to mimic the variation of Li access at different sites of the NMC secondary particle. These approximated boundary conditions naturally induce a global heterogeneity of SoC in the NMC secondary particle. This setting is a simplification of the electrochemical condition at the surface of the NMC secondary particle in contact with the liquid electrolyte, binders, and conductive matrix. The actual surface concentration can be determined by solving the Butler-Volmer equation with an appropriate overpotential profile on the particle surface. To capture the local charge heterogeneity facilitated by intergranular fracture, we couple the diffusion kinetics and damage evolution in the way that Li concentration at the newly formed crack surfaces are set to be the external Li concentration. This is a good replication of the experimental observation that the liquid electrolyte can quickly penetrate into the cracked surface of NMC particles and segregates along the grain boundaries^30^.

**Fig. 4:**
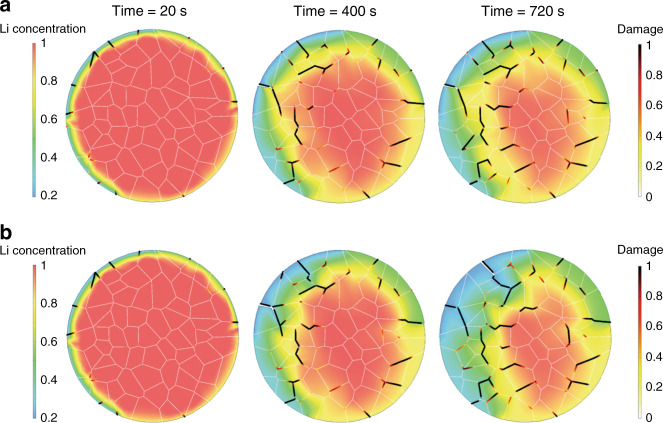
FEM of charge distribution and intergranular fracture in an NMC secondary particle. The evolution of Li concentration and the damage along the grain boundaries without (**a**) and with (**b**) considering the liquid electrolyte penetration.

Figure 4a, b show the difference of Li concentration and the intergranular damage when the penetration of the electrolyte along the cracked surfaces is taken into consideration. Overall, more intergranular cracks are incurred in the regime where a lower concentration boundary condition (higher SoC) is prescribed. The crack distribution is uneven over the particle. The small segments of the interfacial damage tend to interconnect and form major cracks throughout the particle. The progression of mechanical disintegration at the grain boundaries (dark red color in Fig. 4) is driven by (1) the tensile hoop stress in delithiated NMC where the NMC lattice shrinks, and (2) the mismatch strain between the primary particles due to misalignment of their crystalline orientations. The comparison of the Li distribution and damage at the charging times *t* = 400 s and *t* = 720 s exemplifies the corrosion fact of the electrolyte percolation. The results in Fig. 4b show a much higher degree of intergranular damage. In addition, as shown in Supplementary Movie 1, the time evolution of the intergranular fracture along the grain boundaries with considering the liquid electrolyte penetration is visualized in the animation. Local Li starvation at the cracked grain boundaries induces more volumetric change in NMC and therefore more mechanical failure. Meanwhile, the newly formed cracked surfaces provide a fast path of Li transport, which in turn promotes further heterogeneous distribution of Li in the particle. This scenario illustrates the intertwining between the charge heterogeneity and the mechanical damage that the local Li depletion and intergranular cracks promote each other because of the electrolyte penetration in the grain network.

**Fig. 5:**
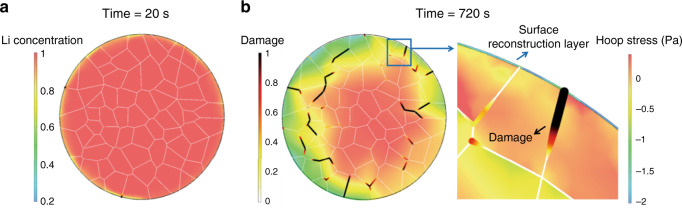
FEM of surface passivation and structural decohesion in an NMC secondary particle. The Li concentration and intergranular damage at two charging times *t* = 20 s (**a**) and *t* = 720 s (**b**). The enlarged inset in **b** shows an overall compressive hoop stress in the passivation layer and an intergranular crack passing through the passivation layer.

We next consider the mutual modulation between surface passivation and intergranular fracture in an NMC secondary particle. Although the surface passivation effect is inhomogeneous over the entire particle (as is demonstrated in our soft X-ray nanoprobe mapping results in Fig. 3a), for illustration purposes, we construct an ideal scenario by building a passivation layer of 20 nm thickness uniformly coated on the NMC particle. The surface phase transition layer in NMC cathode is typically of a rock-salt structure and is inert to Li reaction. The elastic properties of the surface layer are listed in the Supplementary Table S1. We assume that Li diffusivity through the passivation layer is 1% of the Li diffusivity in NMC. Given its small thickness relative to the overall size of the secondary particle, the surface layer provides limited blocking effect on Li diffusion but its presence may reduce the electrolyte penetration into the grain boundaries. Figure 5 shows the Li concentration profile and intergranular damage in the NMC particle at the charging times *t* = 20 s and *t* = 720 s. Compared to the example in Fig. 4, the passivation layer coated particle experiences a much less degree of charge heterogeneity and interfacial damage. A part of the reason is the confinement of the surface layer to the deformation of the NMC particle. Li extraction from NMC induces an overall compressive stress in the surface passivation layer, which reduces the driving force of crack opening along the grain boundaries. It is noteworthy that the severe intergranular damage may also penetrate through the coating layer as shown in Fig. 5b. The other factor is the reduced SoC in the coated NMC, which minimizes the global charge heterogeneity and thus the mechanical damage. The result demonstrates that the surface phase transition layer, which presents mechanical confinement to the inner particle and serves as a barrier of Li transport, alleviates structural damage in the grain structure.

**Fig. 6:**
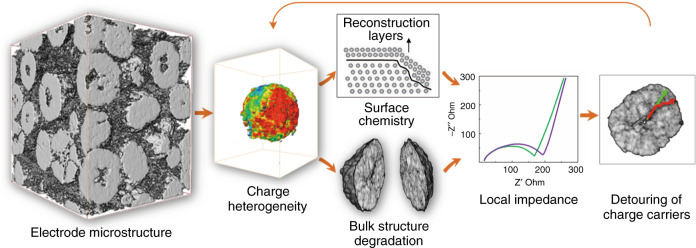
Schematic illustration of the surface-to-bulk mutual modulation. The interplay between the surface chemistry and the bulk microstructure within an individual NMC particle is presented by the arrows (not to scale).

The particle’s bulk porosity is essentially a measurement of the degree of local reaction. This is because the amount of particle fracturing is generally positively associated with the degree of participation in the intercalation/deintercalation^5^. Our experimental results suggest that, within a single secondary particle, the regions that experienced a higher degree of reaction is associated with a more reduced Ni oxidation state on the particle surface, which indicates a more severe local surface reconstruction effect. Based on the experimental observed surface-to-bulk correlation and the insights provided by the FEM results, we formulate a mechanism for the mutual modulation between the surface chemistry and the bulk microstructure, which governs the particle’s response to the reaction driving forces. As shown in Fig. 6, the NMC cathode is populated with structural complexity over a wide range of length scales. These morphological defects are often regarded as the root cause of the heterogeneity in the impedance and charge distribution, which provokes ununiform surface chemical degradation and bulk structure disintegration. The formation of micro cracks and heterogeneous surface passivation would cause further development of the undesired local impedance, which, subsequently, leads to detouring of the charge carriers (Li ions and electrons) and affects the degree of sub-particle level domain utilization. These effects collectively influence the propagation of the reaction fronts and further dictates the charge distribution and evolution. In summary, the surface chemistry and bulk microstructure mutually modulate each other through a complicated mechanism that is closely related with the battery performance.

## Discussion

In this work, we present a direct visualization of the structural and chemical complexity throughout a single NMC811 secondary particle with the combination of nano-resolution X-ray probes in both soft and hard X-ray regimes correlatively. It is observed that the degree of the lattice reconstruction effect is inhomogeneous over the particle surface. More importantly, the regions with higher porosity are associated with more severe surface lattice reconstructions, which suggests a mutual modulation between the surface chemistry and the bulk microstructure. Our FEM results further shed some light on the underlying interplay among the charge heterogeneity, bulk fracture, and surface passivation at the single-particle level. This work presents a fundamental understanding of the coupling effect between the surface chemistry and the bulk microstructure. Such a surface-to-bulk correlation highlights that both crack mitigation and surface modification are key points that shall be considered in an integrated manner for the design of the next-generation cathode materials for LIBs industry. The herein developed correlative nano-resolution imaging method (with both hard and soft X-ray probes) not only paves a new way toward more comprehensive understanding of LIBs material, but also will have profound impacts on a broad range of scientific fields well beyond battery research. We also point out that the battery cathodes are composed of many particles. While the current study has a strong focus on the single-particle level structural and chemical complexity and the intercoupling, a follow-up study of many particles is highly desirable for the electrode level statistical significance. We also clarify that the presented experimental results are at a static state. While the chemical heterogeneity within NMC particles can persist even after long term relaxation of the material and can be utilized as fossil evidence for our investigation of the surface-to-bulk correlation^4^, we acknowledge the importance of utilizing in situ and/or operando methods for studying the dynamic aspect of the reaction experimentally^31–33^. Our study focuses on the surface-to-bulk correlation, which involves a complicated experimental procedure that sets a practical limitation in our experiment. Significant efforts will be needed in the follow-up research in this direction.

## Methods

### Sample preparation

Single NMC811 secondary particle was firstly mounted on top of the W needle with Pt welding (Supplementary Fig. 5). A common sample holder was designed to facilitate efficient sample transfer while keeping the same viewing angle for easier imaging data registration (Supplementary Fig. 6b, d). Such an experimental configuration ensures mechanical stability while providing a good electrical contact for the measurement of the TEY signal over the particle surface. We conducted the correlative X-ray imaging experiment using beamlines 13-1 and 6-2c at Stanford Synchrotron Radiation Lightsource (SSRL, Supplementary Fig. 6c, e), where the scanning soft X-ray nanoprobe and the full-field TXM are installed respectively.

### Electrochemical measurement

The NMC811 was provided by the U.S. Department of Energy’s (DOE) CAMP Facility (Cell Analysis, Modeling and Prototyping) at Argonne National Laboratory. The 1st and 46th cycled electrodes were harvested at charged state (4.5 V) and was cycled using a rate of 0.2 C (for the initial cycle) and 1 C (for the 2nd cycle and beyond), respectively^23^.

### Full-field TXM measurement

The single secondary particle of the charged NMC811 material was mounted on a W needle with a Pt joint using a FEI Helios NanoLab 600i DualBeam FIB/SEM. Nano-tomography of the single secondary particle of the charged NMC811 material was carried out using TXM at beamline 6-2c at SSRL^34^. The Ni K-edge energy 2D map was collected by taking projection images (0.5 s exposure time, 10 repetitions, binning 2, 1024 × 1024 pixels) with scanning the X-ray energy from 8100 to 8800 eV in 134 steps. The repeated exposures were carried out to improve the signal to noise ratio in the images. A typical 2D XANES scan takes about 20 minutes. The nano-tomography data were performed by rotating the sample holder from −90^o^ to 90^o^ with an angular step size of 0.5^o^ and incoming X-ray energy of 8800 eV. A typical single-energy tomographic scan takes about 15–20 minutes. The pixel size is at 34.3 nm at the highest energy (8800 eV) used in our XANES scan. The TXM image’s pixel size varies as a function of the X-ray energy. All the images are scaled to match the data at 8800 eV with pixel size at 34.3 nm. Sample exposure to the air was minimized by using N_2_-filled glove bags for the sample transfer. During the TXM experiment, the sample was placed under a slow and steady helium flow, which has been demonstrated to be effective in protecting the sample from air exposure. The in-house-developed software package named TXM-Wizard was used to perform the data analysis^35^.

### Scanning soft X-ray nanoprobe measurement

Soft X-ray nanoprobe was carried out at beamline 13-1 at SSRL, which is housed in a vacuum chamber and is operated at a pressure of 2 × 10^−8^ mbar. The soft X-ray nanoprobe TEY signal mapping is measured by raster scanning of the sample with a step size of 30 nm and incoming X-ray energies of 854.0 and 856.2 eV, respectively. The scan for a single TEY map using the soft X-ray nanoprobe takes about 40 min at beamline 13-1 of SSRL. The focal size and the scan step of the soft X-ray nanoprobe are both set to 30 nm, which is the nominal spatial resolution of this technique^36^.

### Soft XAS measurement

Soft XAS measurements were conducted at beamline 13-3 at SSRL. The NMC811 electrode was harvested from a conventional coin cell with liquid electrolyte and was dried in order to facilitate soft X-ray measurements in an ultra-high vacuum environment^3^.

### Finite element analysis

We build a more tractable 2D model to simulate the evolution of the Li concentration, stress, and intergranular fracture in NMC during charging. The secondary particle is represented by a circular domain composed of multiple primary particles of random sizes and shapes. The polygonal primary particles are generated using the Voronoi tessellation^37^. The kinetics of Li diffusion is governed by the Fick’s law, $\frac{{\partial c}}{{\partial t}} = [D_{ij}c_{,j}]_{,i}$, where *c* is the Li concentration and *D*_*ij*_ the diffusivity. Li diffusion in NMC is anisotropic. Here we assume that Li diffusivity in the *ab* plane of the NMC lattice is 10 times higher than that along the *c* direction. The initial Li concentration is set to be the maximum concentration in the pristine NMC. A constant concentration boundary condition is prescribed on the particle surface. We set 0.2*C*_max_, 0.3*C*_max_, 0.5*C*_max_, and 0.8*C*_max_ respectively on each quarter of the particle surface to mimic the variation of Li access at different sites of the NMC particle surface. Such variation in real batteries is caused by the incomplete contact of the NMC particles to the electron conduction network or the coverage of the active particles by the polymer binder which has an insulating nature of ion conduction^38^. The delithiation induced strain is calculated as $\varepsilon _L = \frac{{l - l_0}}{{l_0}}$, where *l*_0_ and *l* represent the lattice parameters in the pristine NMC and in the charged NMC at a given state of charge, respectively. Li-extraction induced deformation in NMC is highly anisotropic. The lattice constants of NMC811 in the *a*- and *b*-axis decrease by 2.1% at the fully charged state while the lattice constant in the *c*-axis first increases and then decreases by 3.7% upon the charging voltage of 4.5 V. Here we use the lattice parameters in NMC811 at different charging states measured by Ryu et al.^39^. The deformation kinematics is prescribed by $\varepsilon _{ij} = \frac{1}{2}(u_{i,j} + u_{j,i})$, where *u* represents the displacement field, and the total strain *ε*_*ij*_ includes the elastic strain *ε*_*e*_ and the lithiation/delithiation induced strain *ε*_*L*_. The constitutive law describing the stress-strain relationship is given by $\sigma _{ij} = C_{ijkl}(\varepsilon _e)_{kl}$, where *C*_*ijkl*_ is the elastic constant in the stiffness matrix. Here we consider the anisotropic mechanical property of the NMC lattice^40^. The elastic constants are listed in Supplementary Table 1. The stress field is solved by the equation of momentum equilibrium *σ*_*ij,j*_ = 0. We prescribe a zero-displacement boundary condition at the center of the NMC secondary particle to prevent the rigid motion and a traction-free boundary condition on the particle surface. We use the cohesive zone model to simulate the nucleation and propagation of intergranular cracks. The interfacial failure can be simulated by the progressive damage of the cohesive element layer assigned between the individual primary particles. The damage response of the cohesive element is described by the traction-separation law shown in Supplementary Fig. 7. We define the damage function such that the damage remains zero in the elastic response range and equals to one when the strain energy exceeds the fracture energy. The intermediate value of the damage function is calculated by a linear interpolating function. The governing equations of Li diffusion, deformation kinematics, and cohesive zone model are solved simultaneously at every time step in COMSOL Multiphysics. The built-in time-dependent solver MUMPS (MUltifrontal Massively Parallel sparse direct Solver) is used to solve the co-evolution of Li concentration, stress, and the damage function in the NMC secondary particle. It should be noted that the 2D model is a simplified representation of the NMC secondary particle. The polygonal primary particles generated by the Voronoi tessellation may not capture the entire morphological features in the real samples. In addition, several assumptions are taken in the model, such as Li diffusivity in the surface passivation layer, the ratio of Li diffusivity along the *c*-axis and in the ab plane, and fracture toughness of the intergranular boundaries. Further experimental measurements of the material parameters will provide better input to the theoretical modeling. The model can be further enhanced by considering the difference of Li transport in the bulk and along/across the grain boundaries, and potential regulation of mechanical stresses on the rate of Li diffusion. While the current model can capture the salient features of mutual modulation between charge heterogeneity, bulk fracture, and surface passivation, the simplifications and uncertainties are also worth noting.

## Supplementary information

Supplementary Information

Peer Review File

Description of Additional Supplementary Files

Supplementary Movie 1

## Data Availability

The data that support the plots within this paper and other finding of this study are available from the corresponding author upon reasonable request.
