# Supplementary material for: Mutual modulation between surface chemistry and bulk microstructure within secondary particles of nickel-rich layered oxides
Source: Nat Commun. 2020 Sep 7;11:4433. doi: 10.1038/s41467-020-18278-y (PMC7477569; doi:10.1038/s41467-020-18278-y)
Supplement: Supplementary file 1 — Supplementary Information [file 41467_2020_18278_MOESM1_ESM.pdf]

Supplementary Information for

Mutual modulation between surface chemistry and bulk microstructure within secondary particles of Ni-rich layered oxides

Li *et al.*

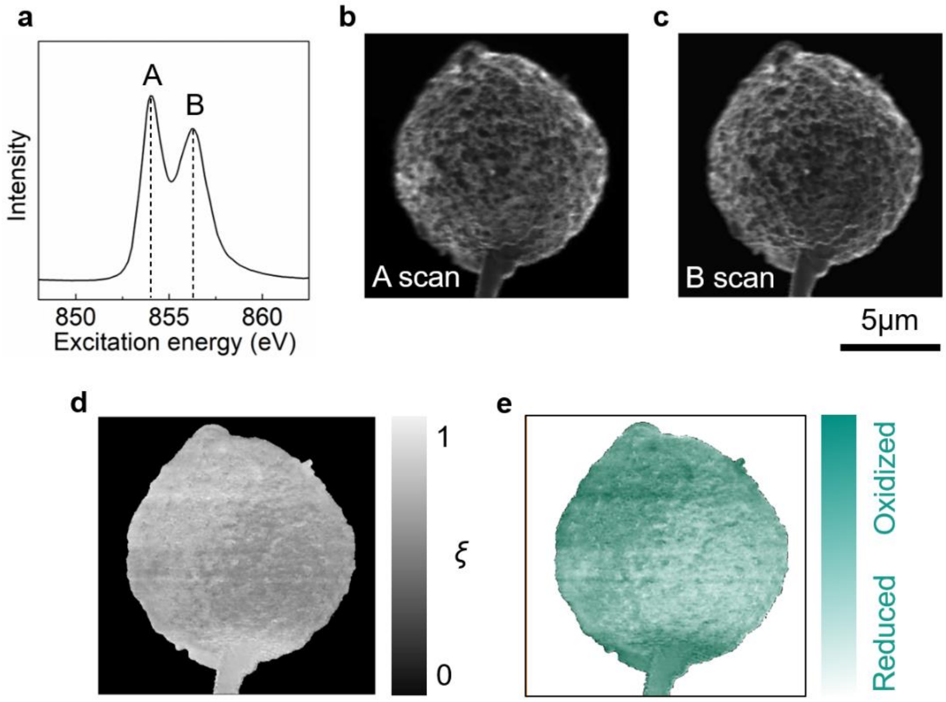

Supplementary Figure 1. Schematic illustration of using scanning soft x-ray nanoprobe to probe the surface Ni valence heterogeneity. **a**, Averaged soft x-ray absorption spectra of the charged NMC811 electrode over the Ni L_3-edge in the TEY mode. A scan (**b**)and B scan (**c**) are scanning soft x-ray nanoprobe TEY signal collected with incoming x-rays energies of 854.0 eV and 856.2 eV, respectively. **d**, TEY intensity ratio (ξ) is calculated using the following equation pixel by pixel: $\xi_{(x,y)} = \frac{I_{856.2}^{\text{TEY}}}{I_{854.0}^{\text{TEY}}+I_{856.2}^{\text{TEY}}}$, in which ($x$, y) is the position of each pixel, I is the TEY intensity at selected incoming energy. **e**, Map of Ni valence state, which was proportional to TEY intensity ratio in (**d**).

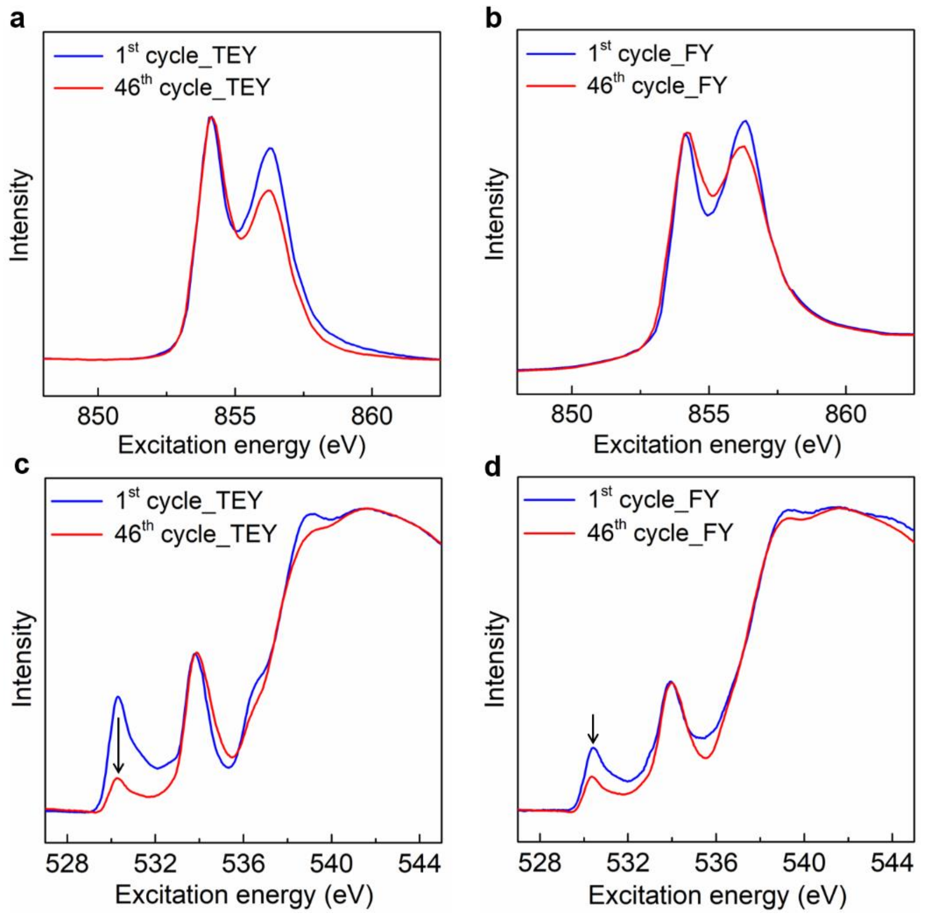

Supplementary Figure 2. XAS spectra of the charged NMC811 electrode. NMC811 electrode measured at the Ni L_3-edge and the O *K*-edge via the TEY mode (**a**, **c**) and the FY mode (**b**, **d**). The electrodes recovered after the 1st cycle (blue) and the 46th cycle (red) are compared to highlight the prolonged electrochemical cycling induced chemical degradation. The black arrows in (**c**) and (**d**) denotes the suppression of the pre-edge peak at excitation energy of ~530.3 eV in oxygen *K*-edge spectra upon electrochemical cycling.

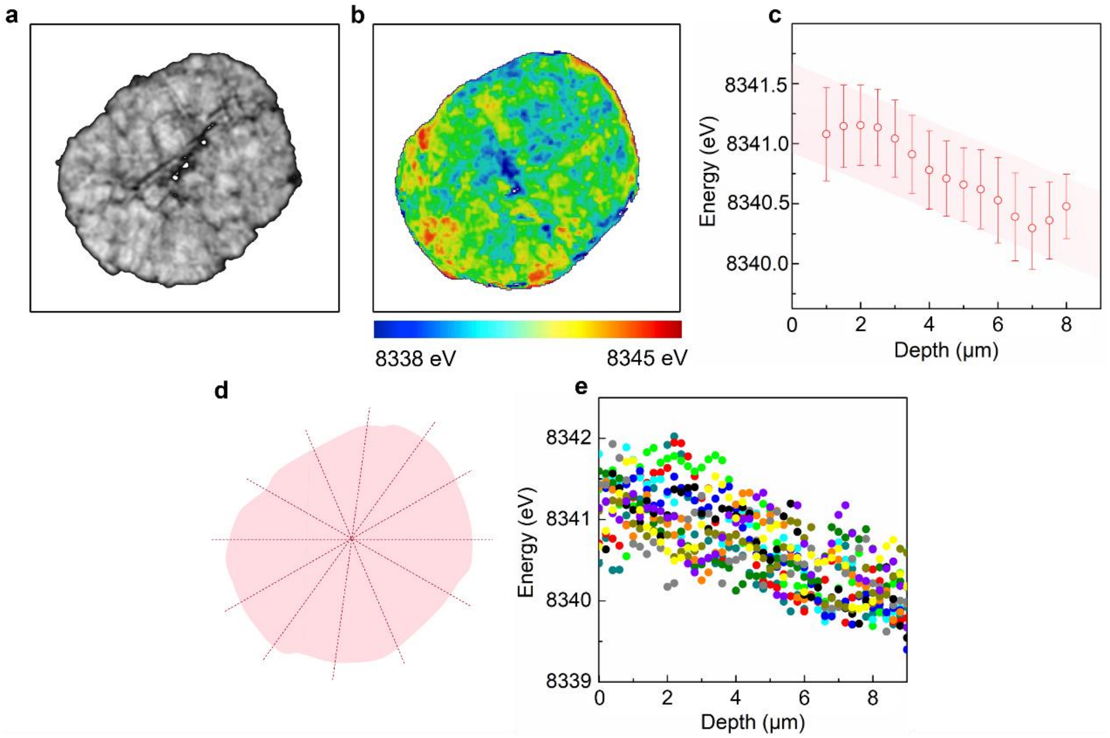

Supplementary Figure 3. Chemical complexity within a charged NMC811 secondary particle. **a**, **b**, The xy-slice through the center of the particle (**a**) and corresponding Ni K-edge energy map (**b**). **c**, The depth profile of the edge energy distribution over the particle. The near-surface regions are generally more oxidized compare to the particle core. **d**, Schematic of the selected lines for plotting the line profiles in (**e**). **e**, Edge energy distribution from the surface to the center over different lines.

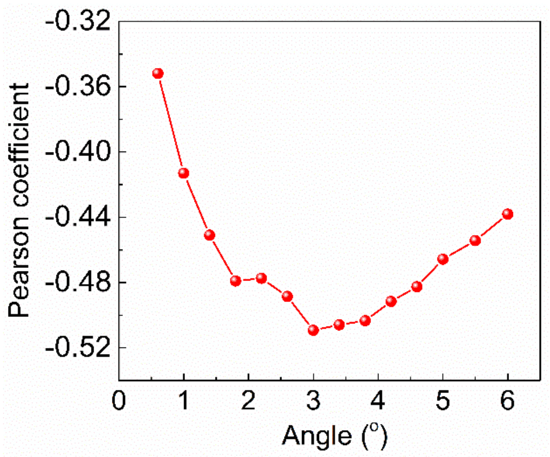

Supplementary Figure 4. The correlation between the surface mapping data and the bulk porosity with different opening angle for the cone-shape regions.

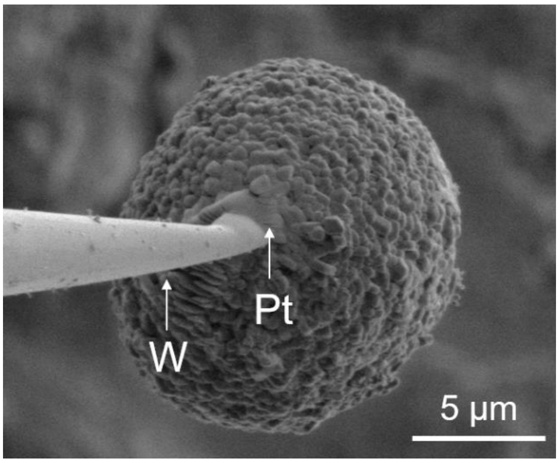

Supplementary Figure 5. SEM image of single NMC811 secondary particle mounted on the W needle with Pt welding.

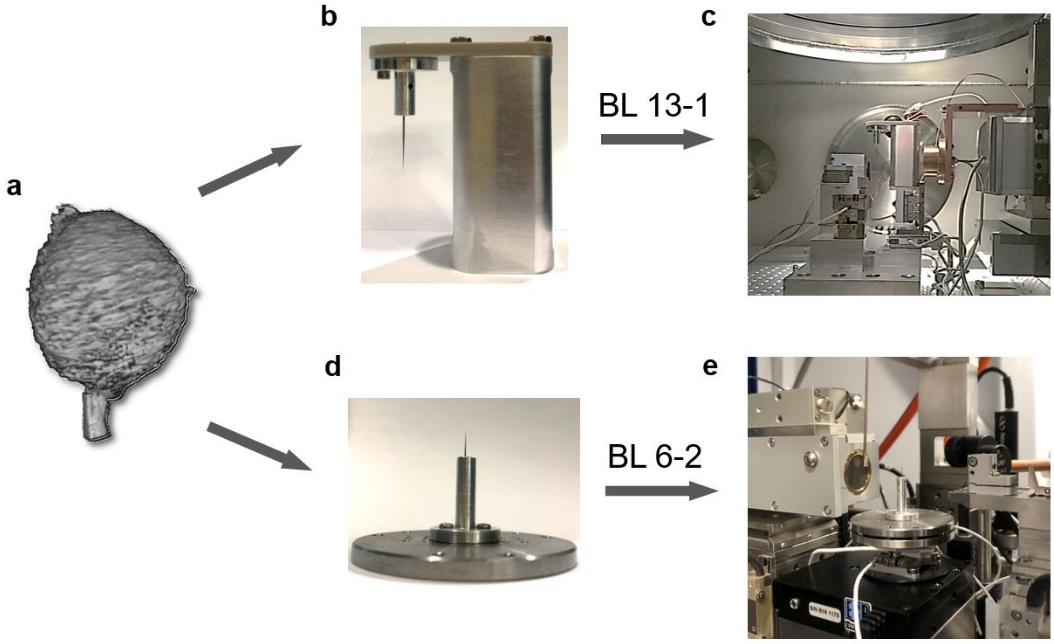

Supplementary Figure 6. Optical photographs of sample holders and beamlines. a, 3D visualization of single NMC811 secondary particle. Optical photographs of the sample holders for BL13-1 (**b**) and 6-2c (**d**). Optical photographs of the sample holders installed on the BL13-1 (**c**) and 6-2c (**e**).

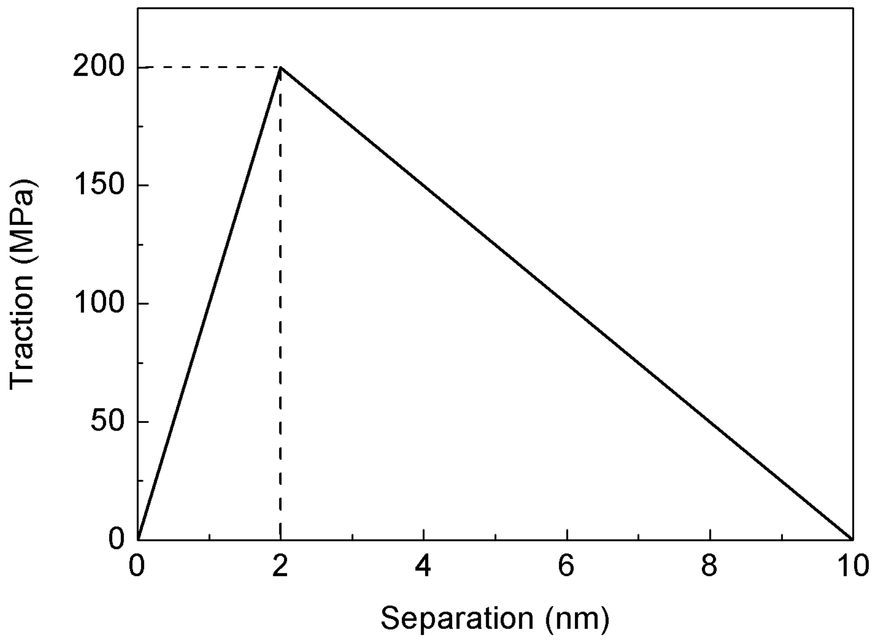

Supplementary Figure 7. The traction-separation law of the cohesive zone model.

The area under the traction-separation curve represents the fracture toughness.

Supplementary Table 1. Parameters used in finite element modeling.

Parameter	Symbol	Value
Particle radius	r	5×10^{-6} m
Elastic constants	C_{11}	259 GPa
	C_{12}	107 GPa
	C_{13}	75 GPa
	C_{33}	194 GPa
	C_{44}	59 GPa
Li diffusivity along *ab* plane	D_{ab}	7×10^{-15} m^2 s^{-1}
Li diffusivity along *c* direction	D_c	7×10^{-16} m^2 s^{-1}
Li diffusivity in surface passivation layer	D_L	7×10^{-17} m^2 s^{-1}
Maximum Li concentration	C_{max}	63887 mol m^{-3}
Tensile strength	t_n	200 MPa
Fracture toughness	G_c	1 J m^{-2}
Elastic modulus of surface passivation layer	E	190 GPa
Poisson's ratio of surface passivation layer	v	0.35
