# Supplementary material for: Mutual modulation between surface chemistry and bulk microstructure within secondary particles of nickel-rich layered oxides
Source: Nat Commun. 2020 Sep 7;11:4433. doi: 10.1038/s41467-020-18278-y (PMC7477569; doi:10.1038/s41467-020-18278-y)
Supplement: Supplementary file 3 — Description of Additional Supplementary Files [file 41467_2020_18278_MOESM3_ESM.pdf]

Description of Additional Supplementary Files

File name: Supplementary Movie 1
Description: The evolution of the intergranular fracture along the grain boundaries with considering the liquid electrolyte penetration
