# Supplementary material for: Mutual modulation between surface chemistry and bulk microstructure within secondary particles of nickel-rich layered oxides
Source: Nat Commun. 2020 Sep 7;11:4433. doi: 10.1038/s41467-020-18278-y (PMC7477569; doi:10.1038/s41467-020-18278-y)
Supplement: Supplementary file 2 — Peer Review File [file 41467_2020_18278_MOESM2_ESM.pdf]

Reviewer #1 (Remarks to the Author):

This is a very strong submission. The authors conduct a detailed morphological and chemical investigation on a single electrode particle with high resolution. The work is novel, presents new understandings of battery electrodes, and outlines a technique that will be very useful for future work. Therefore, I recommend publication after some minor changes.
Around line 53 – recent work by NREL using XRDCT saw some depth- and lithiation-dependent compositions and crystallographic structures in LMO electrodes (https://doi.org/10.1038/s41467-020-14467-x). Perhaps worth mentioning in introduction.
Fig 1 – TEY is defined after placement of figure
Figure 1c – what area are the labelled regions referring to?
Figure 3 – please make it clear in the caption what the symbol on secondary y axis in c represents. Also, I'm not sure how to interpret the line from A to B – some uncertainty: is this a line of voxels in 3D space? is there some binning or differences in resolution between the techniques? Should the x axis have units of distance in c? Some extra clarity on this would be useful to readers. I'm confused by how the soft x ray technique reached the core regions. Similar for d, are the datapoints based on voxels and how was the valence state know for those specific voxel volumes? Am I correct it thinking that for each volume measurement of valence states, there would be a distribution of states rather than a single value? How does that affect the measurement?
Some discussion on limitations/uncertainty in the models would be useful – e.g. transport at/along grain boundaries, single-direction Li transport in single crystals, and static boundaries (rather than moving boundaries with volume expansion).
Physically, I don't understand why there would be a correlation between porosity of the cone region and the valence state. The authors hypothesize that microstructure features influence activity at the surface, which makes some sense, but the radial connection between surface to bulk may not be the best. For example, a surface grain may be oriented such that Li transports in a more circumferential direction than radial, thus the surface properties there may not be linked to morphological properties radially inwards from that point. It's an interesting hypothesis, but it's worth discussing the uncertainties of this hypothesis. Discussions like this make manuscripts even more rich, in my opinion.
Methods: Please include more info on:
- Exposure and data acquisition times.
- Estimated spatial resolutions of the techniques.
- Some more info on the environment used for the single particle.

Reviewer #2 (Remarks to the Author):

The manuscript presents an impressive combination of high-resolution transmission X-ray imaging and CT as well as X-ray spectroscopy. However, the static, end of test, air-exposedvary imaging is less than ideal for validating the model results.

The manuscript uses finite element modeling of the chemo-mechanical behavior including crack propagation to understand the X-ray imaging. The main novelty in the modeling is the heterogeneous surface conditions (boundary conditions) imposed to mimic the valence state heterogeneity in the X-ray spectroscopy. This is applied with different current boundary conditions rather than incorporating different chemical/kinetic properties at the surface. Thus, the modeling is more heuristic than would be preferred.

The main issue concern with this work is what appears to be a tenuous connection between the imaged particle and the simulation. The concern is because of the static imaging and the dynamic modeling and assigning the static observations to the dynamic simulations. Without knowing the beginning of life or initially discharged state of the particle, it is questionable whether conclusions should be drawn from images of a single particle.

Was just the one particle imaged for each state, or is it just a representative example of an ensemble?

Is it possible that Pt welding for the sample mount of the particle impacted the observed valence state? The difference in the surface valence seems to vary with distance from the weld.

Line 289: Where does the assumption of 1% Li diffusivity in the passivation layer come from? Is there data to support this, or is it just a modeling assumption?

The dynamic fracture processes the model predicts is not evident in the imaging.

Various typos, misspellings, and grammatical errors need to be corrected. The manuscript needs some careful editing:
Line 29 “severer”
Line 37 “LIB” should be “LIBs”

Point-to-point revision summary for

Article NCOMMS-20-16190 *Mutual modulation between surface chemistry and bulk microstructure within individual secondary particles of Ni-rich layered oxides*

Reviewer #1:

This is a very strong submission. The authors conduct a detailed morphological and chemical investigation on a single electrode particle with high resolution. The work is novel, presents new understandings of battery electrodes, and outlines a technique that will be very useful for future work. Therefore, I recommend publication after some minor changes.

Response:

We thank the reviewer for the positive assessment and for recommending the publication of our manuscript in *Nature Communications*.

1. Around line 53 – recent work by NREL using XRDCT saw some depth- and lithiation-dependent compositions and crystallographic structures in LMO electrodes (https://doi.org/10.1038/s41467-020-14467-x). Perhaps worth mentioning in introduction.

Response:

We thank the reviewer for bringing relevant literature to our attention. We have revised the introduction section accordingly. We would like to highlight that, although the size of the x-ray focal spot (i.e. the nominal spatial resolution) for the XRDCT technique is not very small, this technique is measuring the x-ray diffraction signal that is related to the material's lattice structure and, thus, is sensitive to the atomic-scale structural and chemical evolution of the cathode material upon electrochemical cycling.

Revision:

In the 2nd paragraph of the introduction, we have added the following discussions and the corresponding reference as suggested.

"It is worth mentioning that the operando high-resolution X-ray diffraction-computed

tomography (XRDCT) has been demonstrated as a promising approach to quantify crystallographic heterogeneities spatially and temporally within and between the electrode particles [Nat. Commun. 11, 631 (2020)]. We would like to highlight that, although the size of the x-ray focal spot (i.e. the nominal spatial resolution) for the XRDCT technique may not be very fine, this technique is sensitive to the material's lattice structure, which is directly related with the atomic-scale structural and chemical evolution of the cathode material upon electrochemical cycling."

2. Fig 1 – TEY is defined after placement of figure

Response:

In the revised manuscript, we provided the definition of total electron yield (TEY) in **Fig. 1** and removed this definition in the 2nd paragraph of the results section.

3. Figure 1c – what area are the labelled regions referring to?

Response:

Fig. 1c is now revised with the labels for regions 1 and 2 included (also see **Fig. R1**c below).

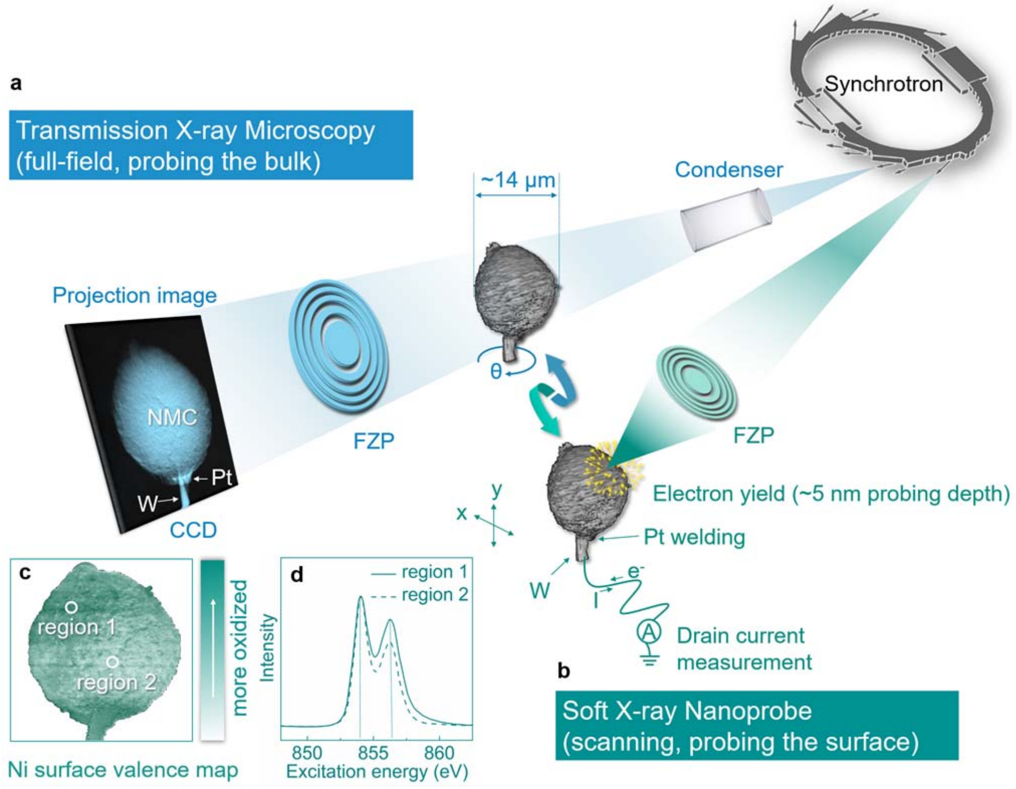

Fig. R1 Schematic illustration of the correlative imaging for a single NMC811 secondary particle. In the full-field TXM measurement (**a**, light blue color), the transmission images of the particle were acquired as the sample is rotated in a tomographic scan. In the soft x-ray nanoprobe measurement (**b**, dark green color), the total electron yield (TEY) signal is recorded by measuring the drain current. Through raster scanning the particle with respect to the focal point, the surface chemical inhomogeneity (**c**) is revealed based on the energy dependent local TEY signal (**d**) over different surface regions (as labeled in (**c**)).

4. (1) Figure 3 – please make it clear in the caption what the symbol on secondary y axis in c represents. (2) Also, I'm not sure how to interpret the line from A to B – some uncertainty: is this a line of voxels in 3D space? (3) is there some binning or differences in resolution between the techniques? (4) Should the x axis have units of distance in c? Some extra clarity on this would be useful to readers.

Response:

Following the reviewer's suggestion, we have added more information about this figure in the manuscript.

(1) For **Fig. 3**c, the ξ on secondary y axis represents relative Ni valence state, which is the ratio between the TEY intensity at 856.2 eV and the sum of that at 854.0 eV and 856.2 eV pixel by pixel. The definition of ξ was provided in the text in our previous submission and we add it to the figure caption as well in the revised version.

(2) The line from A to B denotes a single-pixel-wide line in the projected 2D images and the length of this line is approximately the diameter of the particle. Over this line, we plot the intensity profiles based on the surface valence mapping (**Fig. 3**a) and the bulk porosity mapping (**Fig. 3**b) data, respectively.

(3) The nominal spatial resolution of the soft x-ray nanoprobe and that of hard x-ray TXM in our experiment are very close (both at ~ 30 nm). However, the exact pixel sizes are slightly different in these two measurements (30 nm for the soft x-ray nanoprobe and 34.3 nm for hard x-ray TXM) and we rescaled the matrix slightly (using bilinear interpolation) to match the pixels for one-to-one comparison. It is useful to point out that, because the pixel sizes of data from these two measurements are rather close, the rescaling of the matrix does not have a substantial impact on the data quality.

(4) In the revised manuscript, we added the units of distance for x axis in **Fig. 3c**.

Revision:

In the caption of **Fig. 3**, we have added the discussion about the ξ.

"The ξ is the ratio between the TEY intensity at 856.2 eV and the sum of that at 854.0 eV and 856.2 eV pixel by pixel, which represents relative Ni valence state."

In the 3rd paragraph of the "The correlation between local surface chemistry and bulk microstructure" section, we have added the following discussion.

"The line from A to B denotes a single-pixel-wide line in the projected 2D images. Over this line, we plot the intensity profiles based on the surface valence mapping and the bulk porosity mapping data, respectively. The pixel sizes of the data from the two imaging methods are very close but not identical. We utilized the bilinear

interpolation method to rescale the matrix slightly for a pixel-to-pixel matching."

5. I'm confused by how the soft x ray technique reached the core regions. Similar for Fig. 3d, are the datapoints based on voxels and how was the valence state know for those specific voxel volumes? Am I correct it thinking that for each volume measurement of valence states, there would be a distribution of states rather than a single value? How does that affect the measurement?

Response:

The probing depth of the soft x-ray nanoprobe (with lateral resolution at ~30 nm) in TEY mode is around 5 nm, which cannot reach the particle's core regions. While this characteristic of soft x-rays limits its capability of probing the signal in the bulk, it certainly enhances the soft x-ray nanoprobe's sensitivity to the surface chemistry to a level that is well beyond the resolution limit of the hard x-ray TXM. The key idea here is to combine the advantages of both techniques by conducing these measurements on the same particle in a correlative manner. While the experimental data from the soft x-ray nanoprobe naturally confine (in depth) on the particle surface, we numerically project the bulk porosity (the hard x-ray TXM data) to the particle surface (see **Fig. 2**b-2d) for a one-to-one lateral comparison. After careful registration and rescaling of these two maps, we correlate the surface valence states and the bulk porosity (numerically projected to the particle surface) pixel by pixel over the particle surface (not the bulk). It should be noted that the local Ni valence state of every surface pixel is represented by ξ (see definition given in the caption of **Fig. 3**d), which is the ratio between the TEY intensity at 856.2 eV and the sum of that at 854.0 eV and 856.2 eV.

We would like to point out that there could be redox heterogeneity at a length scale finer than our spatial resolution (~30 nm). If we interpret the question from the reviewer correctly, this is the "distribution of states" within each unit volume (e.g. voxel) that the reviewer referred to. Due to the limited spatial resolution and the nature of our techniques, we are not able to reveal the sub-pixel level chemical heterogeneity. In our soft x-ray nanoprobe data, a surface pixel is represented by one

number, which is the averaged valence state over the corresponding surface pixel. There are some imaging methods that could potentially offer sub-pixel level information. For example, in the XRDCT technique [Nat. Commun. 11, 631 (2020)], after measuring the local XRD pattern associated with a certain pixel, it is possible to refine the XRD data to evaluate the broadening of the diffraction peaks, which correlates with the sub-pixel lattice disordering. The x-ray tensor tomography is another method that could offer sub-pixel level information by evaluating the critical feature size and orientation from the small angle scattering pattern [Nature 527, 353-356 (2015)]. These imaging methods can offer valuable information for the study of battery materials. We have added some discussions along this line in the revised manuscript.

Revision:

In the 5th paragraph of the "The correlation between local surface chemistry and bulk microstructure" section, we have added the following discussions.

"We would like to point out that there could be redox heterogeneity at a length scale finer than our spatial resolution (~30 nm). Due to the limited spatial resolution and the nature of our techniques, we are not able to reveal the sub-pixel level chemical heterogeneity. In our soft x-ray nanoprobe data, a surface pixel is represented by one number, which is the averaged valence state over the corresponding surface pixel. There are some imaging methods that could potentially offer sub-pixel level information. For example, in the XRDCT technique [Nat. Commun. 11, 631 (2020)], after measuring the local XRD pattern associated with a certain pixel, it is possible to refine the XRD data to evaluate the broadening of the diffraction peaks, which correlates with the sub-pixel lattice disordering. The x-ray tensor tomography is another method that could offer sub-pixel level information by evaluating the critical feature size and orientation from the small angle scattering pattern [Nature 527, 353-356 (2015)]. These imaging methods can also offer valuable information for the study of battery materials."

6. Some discussion on limitations/uncertainty in the models would be useful – e.g.

transport at/along grain boundaries, single-direction Li transport in single crystals, and static boundaries (rather than moving boundaries with volume expansion).

Response:

We appreciate this comment and would like to make a few clarifications regarding our modeling effort. We consider anisotropic diffusion of Li, anisotropic deformation, and anisotropic mechanical properties of NMC. Details were described in the manuscript. The NMC secondary particle is represented as an aggregation of single-crystalline primary particles of random sizes and grain orientations.

The single crystal model would not be very relevant to the secondary NMC particles. The reviewer might refer to our earlier work "K. Zhao, et al. Fracture of electrodes in lithium-ion batteries caused by fast charging. Journal of Applied Physics. 108, 073517 (2010)" for the finite element modeling of single crystal $LiCoO_2$ particle with 2D diffusion of Li in the *ab* plane.

The static boundary condition would be a simplified model. Given the small deformation (~5% volumetric strain upon fully charge) of NMC, a simplified model of static boundary conditions will not cause significant difference as compared to the results of the moving boundary condition we used. Nevertheless, the effect of the geometric nonlinearity will be substantial for the large-deformation electrodes such as the insertion-type and conversion-type materials. A theoretical framework considering the large deformation and inelasticity can be found in the following paper: "K. Zhao, et al. Large plastic deformation in high-capacity lithium-ion batteries caused by charge and discharge. Journal of the American Ceramic Society. 94, S226-S235 (2011)".

Li transport along the grain boundary will be a great feature to consider. In the current model we only consider Li segregation along intergranular boundaries when the liquid electrolyte infiltrates into the boundaries of primary particles. In an ongoing work, we are calculating Li diffusivity along different types of grain boundaries in NMC. Preliminary results are that the activation energy of Li diffusion is highly dependent on the nature of the grain boundary – Li diffusivity along and across the grain boundaries can vary by several orders of magnitude depending on the local chemical

environment.

Revision:

Following the reviewer's suggestion, we have added the following discussions in the 6th paragraph of the methods, the corresponding discussion has added in the revised manuscript.

"It should be noted that the 2D model is a simplified representation of the NMC secondary particle. The polygonal primary particles generated by the Voronoi tessellation may not capture the entire morphological features in the real samples. In addition, several assumptions are taken in the model, such as Li diffusivity in the surface passivation layer, the ratio of Li diffusivity along the c-axis and in the ab plane, and fracture toughness of the intergranular boundaries. Further experimental measurements of the material parameters will provide better input to the theoretical modeling. The model can be further enhanced by considering the difference of Li transport in the bulk and along/across the grain boundaries, and potential regulation of mechanical stresses on the rate of Li diffusion. While the current model can capture the salient features of mutual modulation between charge heterogeneity, bulk fracture, and surface passivation, the simplifications and uncertainties are also worth noting."

7. Physically, I don't understand why there would be a correlation between porosity of the cone region and the valence state. The authors hypothesize that microstructure features influence activity at the surface, which makes some sense, but the radial connection between surface to bulk may not be the best. For example, a surface grain may be oriented such that Li transports in a more circumferential direction than radial, thus the surface properties there may not be linked to morphological properties radially inwards from that point. It's an interesting hypothesis, but it's worth discussing the uncertainties of this hypothesis. Discussions like this make manuscripts even more rich, in my opinion.

Response:

We fully agree with the reviewer that the realistic Li diffusion pathways in a

secondary NMC particle is not simply radial. This is because the randomly oriented primary grains in the secondary particle not only define the intra-grain optimal directions for Li diffusion, but also induce ubiquitous grain boundaries and structural complexities that further complicate the heterogeneous and anisotropic energy barriers for the inter-grain Li diffusion. The lattice defects including the local short-range ordering can also impact the local resistance to the Li diffusion [Chem 4, 2108-2123 (2018)]. In addition to the particles' intrinsic structural complexities, the in-situ formed particle cracks can cause liquid electrolyte infiltration, forming new solid-electrolyte interphase that could facilitate the Li-ion transportation [Nano Energy 53, 753-762 (2018)]. We believe this is one of the fundamental reasons for the observed weak correlation between the surface degradation and bulk porosity.

However, from a statistical perspective, the outward Li diffusion is unambiguous. This is evidenced by the depth dependent redox profile that has been observed in many NMC secondary particles at different states of charge, regardless of the degree of particle cracking (see examples in **Fig. R2** and Supplementary **Fig. 3**, this is also reported in our recent publication [Nat. Commun. 11, 83 (2020)]).

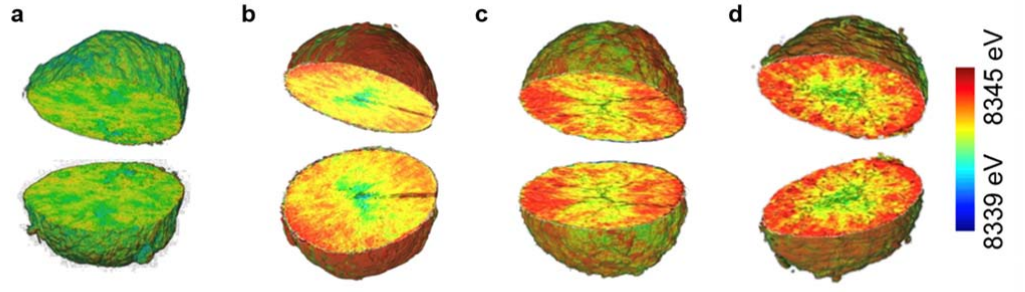

Fig. R2. 3D XANES mapping over the Ni K-edge for a few randomly selected NMC particles. The first particle (**a**) is at the pristine state and the rest of them are charged to different voltages (**b-d**). Regardless the degree of particle cracking, a depth dependent oxidation state distribution is clearly observed in the three charged particles, in which the surface regions are relatively more oxidized and the core regions are relatively more reduced. This observation suggests the overall outward Li diffusion, upon charging to high voltage. (unpublished data)

With these considerations, it is useful to construct a balanced approach for evaluating the surface-to-bulk interplay. Therefore, in our approach, we adjust the size of the bottom of the cone by changing the opening angle (0.6-6°) to reflect the degree of the tortuosity in the Li-ion's outward diffusion pathways. When the opening angle is set to relatively small, it indicates a more radical and less torturous outward Li diffusion. As shown in **Fig. R3**, an optimal opening angle of 3° is selected by evaluating the degree of correlation between the directly measured soft x-ray nanoprobe surface mapping data and the numerically projected bulk porosity onto the particle surface. In our revision, we have added some discussion along this line.

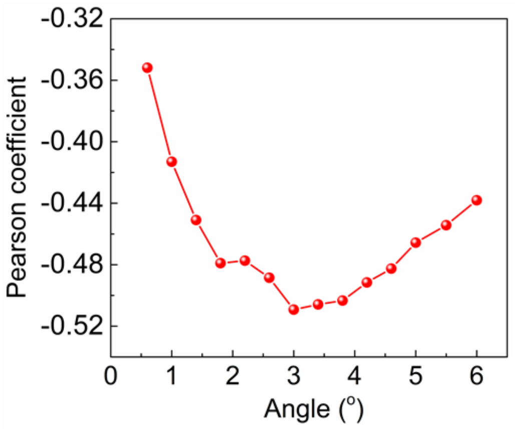

Fig. R3. The correlation between the surface mapping data and the bulk porosity with different opening angle for the cone-shape regions.

Revision:

In the 4th paragraph of the "The correlation between local surface chemistry and bulk microstructure" section, we have added the following discussion.

"It should be noted that the Li-ion's diffusion pathways within a secondary NMC particle is rather complicated. This is because the randomly oriented primary grains in the secondary particle not only define the intra-grain optimal diffusion directions for Li-ions, but also induce ubiquitous grain boundaries and structural complexities that further complicate the heterogeneous and anisotropic energy barriers for the

inter-grain Li-ion diffusion. The lattice defects including the local short-range ordering can also affect the local resistance for the Li diffusion [Chem 4, 2108-2123 (2018)]. In addition to the particles' intrinsic structural complexities, the in-situ formed particle cracks can cause liquid electrolyte infiltration, forming new solid-electrolyte interphase that could facilitate the Li-ion transportation in the short term but build-up impedance over the long run [Nano Energy 53, 753-762 (2018)]. When projecting the bulk porosity to the particle surface using the methodology described in Fig. 2, it is important to take the above discussed complexity into consideration and choose the cone volume with properly selected shape. Therefore, in our approach, we adjust the size of the bottom of the cone by changing the opening angle (0.6-6°) to reflect the degree of the tortuosity in the Li-ion's outward diffusion pathways. When the opening angle is set to relatively small, it indicates a more radical and less torturous outward Li diffusion. As shown in Supplementary Fig. 4, an optimal opening angle of 3° is selected by evaluating the degree of correlation between the directly measured soft x-ray nanoprobe surface mapping data and the numerically projected bulk porosity onto the particle surface. We acknowledge that engineering of the NMC secondary particles with purposely aligned primary grains could impact our results and our model needs to be adjusted accordingly in those cases [Nat. Commun. 11, 83 (2020)]."

8. Methods: Please include more info on:

(1) Exposure and data acquisition times.

(2) Estimated spatial resolutions of the techniques.

(3) Some more info on the environment used for the single particle.

Response:

Following the reviewer's suggestion, we have added the following information in the methods section in the revised manuscript.

(1) Exposure and data acquisition times.

The Ni K-edge energy 2D map was collected by taking projection images (0.5 s exposure time, 10 repetitions, binning 2, 1024 × 1024 pixels) with scanning the x-ray

energy from 8100 to 8800 eV in 134 steps. The repeated exposures were carried out to improve the signal to noise ratio in the images. A typical 2D XANES scan takes about 20 minutes. The nano-tomography data were performed by rotating the sample holder from -90° to 90° with an angular step size of 0.5^o and incoming x-ray energy of 8800 eV. A typical single-energy tomographic scan takes about 15-20 minutes. The soft x-ray nanoprobe TEY signal mapping is measured by raster scanning of the sample with a step size of 30 nm and incoming x-ray energies of 854.0 eV and 856.2 eV, respectively. The scan for a single TEY map using the soft x-ray nanoprobe takes about 40 minutes at beamline 13-1 of SSRL.

(2) Estimated spatial resolutions of the techniques.

The focal size and the scan step of the soft x-ray nanoprobe are both set to 30 nm, which is the nominal spatial resolution of this technique. For the hard x-ray TXM, the pixel size is 34.3 nm at the highest energy (8800 eV) used in our XANES scan. The TXM image's pixel size varies as a function of the x-ray energy. All the images are scaled to match the data at 8800 eV with pixel size of 34.3 nm.

(3) Some more information on the environment used for the single particle.

The soft x-ray nanoprobe is housed in a vacuum chamber and is operated at a pressure of 2 × 10^{-8} mbar. It should be noted that we conducted the soft x-ray nanoprobe mapping prior to the hard x-ray nano-tomography on the same particle. This is because the surface chemical mapping using soft x-ray nanoprobe is more sensitive to the sample environment (e.g. potential air exposure). Sample exposure to the air was minimized by using N_2-filled glove bags for the sample transfer. During the TXM experiment, the sample was placed under a slow and steady helium flow, which has been demonstrated to be effective in protecting the sample from the air exposure.

Revision:

In the 3rd paragraph of the methods, the corresponding discussion has added in the revised manuscript.

"The Ni K-edge energy 2D map was collected by taking projection images (0.5 s exposure time, 10 repetitions, binning 2, 1024 × 1024 pixels) with scanning the x-ray energy from 8100 to 8800 eV in 134 steps. The repeated exposures were carried out

to improve the signal to noise ratio in the images. A typical 2D XANES scan takes about 20 minutes. The nano-tomography data were performed by rotating the sample holder from -90° to 90° with an angular step size of 0.5^o and incoming x-ray energy of 8800 eV. A typical single-energy tomographic scan takes about 15-20 minutes. The pixel size is at 34.3 nm at the highest energy (8800 eV) used in our XANES scan. The TXM image's pixel size varies as a function of the x-ray energy. All the images are scaled to match the data at 8800 eV with pixel size at 34.3 nm. Sample exposure to the air was minimized by using N_2-filled glove bags for the sample transfer. During the TXM experiment, the sample was placed under a slow and steady helium flow, which has been demonstrated to be effective in protecting the sample from the air exposure."

In the 4th paragraph of the methods, the corresponding discussion has added in the revised manuscript.

"Soft x-ray nanoprobe was carried out at beamline 13-1 at SSRL, which is housed in a vacuum chamber and is operated at a pressure of 2 × 10^{-8} mbar. The soft x-ray nanoprobe TEY signal mapping is measured by raster scanning of the sample with a step size of 30 nm and incoming x-ray energies of 854.0 eV and 856.2 eV, respectively. The scan for a single TEY map using the soft x-ray nanoprobe takes about 40 minutes at beamline 13-1 of SSRL. The focal size and the scan step of the soft x-ray nanoprobe are both set to 30 nm, which is the nominal spatial resolution of this technique."

Reviewer #2:

1. The manuscript presents an impressive combination of high-resolution transmission X-ray imaging and CT as well as X-ray spectroscopy. However, the static, end of test, air-exposed vary imaging is less than ideal for validating the model results.

Response:

We thank the reviewer for this comment. We agree with the reviewer that the in-situ/*operando* imaging results could potentially offer more valuable insights. This is indeed one of the research frontiers that we are actively pursuing [ACS Energy Lett. 2, 1240-1245 (2017)]. The in-situ visualization of the structural and chemical evolutions within battery materials has been demonstrated using hard x-ray tools [Science 342, 716-720 (2013); Nat. Commun. 9, 2340 (2018); Nat. Commun. 7, 12372 (2016); Nat. Commun. 6, 6924 (2015); Energy Environ. Sci. 7, 2771-2777 (2014)]. We acknowledge that there are also several impressive efforts of utilizing soft x-ray tools for in-situ/operando study of energy materials [Science 353, 566-571 (2016); Nat. Commun. 6, 6097 (2015); Nat. Commun. 4, 2568 (2013)]. However, for the soft x-ray nanoprobe, it remains very challenging to conduct the in-situ experiments, particularly, for the detection of the total electron yield (TEY) signals in a spatially resolved fashion.

In the current work, the scientific goal is to reveal the surface-to-bulk interplay within an individual battery cathode particle. As the reviewer has hinted, such interplay has a dynamic nature and could evolve over time. We fully agree with the reviewer that an in-situ/operando experiment would be ideal.

There are, however, very significant practical constrains in our experiment. First, we choose to the use the soft x-ray nanoprobe for measuring the surface heterogeneity due to its surface sensitivity (TEY signal with 5 nm probing depth) and sufficient lateral spatial resolution (x-ray focal spot at ~30 nm). As discussed above, it is already a frontier challenge to measure the spatially resolved TEY signal in an in-situ experiment. Second, it is important for us to correlatively combine the soft x-ray nanoprobe measurement with the hard x-ray nano-tomography, which is available at a different beamline, for gathering both the surface and the bulk information of the

same particle. To our knowledge, the correlative soft and hard x-ray nano-resolution imaging results presented in our current paper is unprecedented. We would also point out the chemical heterogeneity within NMC particles can persist even after long term relaxation of the material [Adv. Mater. 28, 6631-6638 (2016)]. Therefore, it is reasonable for us to use the particle at a static state as fossil evidence for investigating the surface-to-bulk correlation. An in-situ/operando experiment of this kind is very challenging and valuable. Significant efforts will be needed in the follow-up research in this direction.

Finally, we would like to address the comment regarding the air exposure. We are fully aware of the importance to avoid sample exposure to the air and have carried out our best possible practice in the beamline experiments. The soft x-ray nanoprobe is housed in a vacuum chamber and is operated at a pressure of 2×10^{-8} mbar, which avoid the possible air-exposed risk during the measurement. We conducted the soft x-ray nanoprobe mapping prior to the hard x-ray nano-tomography on the same particle. This is because the surface chemical mapping using soft x-ray nanoprobe is more sensitive to the sample environment. In addition, sample exposure to the air was minimized by using N_2-filled glove bags for the sample transfer. During the TXM experiment, the sample was placed under a slow and steady helium flow, which has been demonstrated to be effective in protecting the sample from the air exposure. These details are now included in the method section for clarification.

Revision:

We have added the following discussions in the discussion section.

"We also clarify that the presented experimental results are at a static state. While the chemical heterogeneity within NMC particles can persist even after long term relaxation of the material and can be utilized as fossil evidence for our investigation of the surface-to-bulk correlation [Adv. Mater. 28, 6631-6638 (2016)], we acknowledge the importance of utilizing in-situ and/or operando methods for studying the dynamic aspect of the reaction experimentally [Science 342, 716-720 (2013); Nat. Commun. 9, 2340 (2018), Science 353, 566-571 (2016)]. Our study focuses on the surface-to-bulk correlation, which involves a complicated experimental procedure

that sets a practical limitation in our experiment. Significant efforts will be needed in the follow-up research in this direction."

In the 3rd paragraph of the methods, the corresponding discussion about the sample environment during the experiment has added in the revised manuscript.

"Sample exposure to the air was minimized by using N_2-filled glove bags for the sample transfer. During the TXM experiment, the sample was placed under a slow and steady helium flow, which has been demonstrated to be effective in protecting the sample from the air exposure."

In the 4th paragraph of the methods, the corresponding discussion has added in the revised manuscript.

"Soft x-ray nanoprobe was carried out at beamline 13-1 at SSRL, which is housed in a vacuum chamber and is operated at a pressure of 2×10^{-8} mbar."

2. The manuscript uses finite element modeling of the chemo-mechanical behavior including crack propagation to understand the X-ray imaging. The main novelty in the modeling is the heterogeneous surface conditions (boundary conditions) imposed to mimic the valence state heterogeneity in the X-ray spectroscopy. This is applied with different current boundary conditions rather than incorporating different chemical/kinetic properties at the surface. Thus, the modeling is more heuristic than would be preferred.

Response:

We appreciate the very encouraging comment. As the reviewer correctly pointed out, one of the features of FEA is that we prescribe different concentration boundary conditions in different domains of the particle surface to mimic the variation of Li access at different sites of the NMC secondary particle. This approximated boundary condition induces a *global heterogeneity* of SoC in the NMC secondary particle. To capture the *local charge heterogeneity,* we focus on the simulation of Li transport along the grain boundaries facilitated by intergranular fracture. This part is to replicate the experimental observation that liquid electrolyte can quickly penetrate the cracked surface of NMC particles and segregates along the grain boundaries.

We fully agree with the reviewer that a more realistic boundary condition can be determined by solving the Butler-Volmer equation at the interface between the NMC particle and the liquid electrolyte with an appropriate overpotential profile. This approach will require a careful examination of the local contact of the NMC particle with the surrounding binder and conductive matrix, and require an implementation of the coupled electrochemical kinetics and mechanics equations. While we are aware of this refined approach, we took a simplified model to capture and understand the major feature of mutual modulation between charge heterogeneity, bulk fracture, and surface passivation observed in the experiments. We have added the following discussions in the computational part to reflect the reviewer's comment. If the reviewer permits, we will pursue the refined model with an incorporation of the electrochemical kinetics in the next work.

Revision:

We have added the following discussions in the 2nd paragraph of "Theoretical insight into the surface-to-bulk mutual modulation" section.

"These approximated boundary conditions naturally induce a global heterogeneity of SoC in the NMC secondary particle, but are simplification of the electrochemical kinetics at the surface of the NMC secondary particle in contact with the liquid electrolyte, binders, and conductive matrix. The actual surface concentration can be determined by solving the Butler-Volmer equation with an appropriate overpotential profile on the particle surface."

We also added the following discussions in the 6th paragraph of methods.

"It should be noted that the 2D model is a simplified representation of the NMC secondary particle. The polygonal primary particles generated by the Voronoi tessellation may not capture the entire morphological features in the real samples. In addition, several assumptions are taken in the model, such as Li diffusivity in the surface passivation layer, the ratio of Li diffusivity along the c-axis and in the ab plane, and fracture toughness of the intergranular boundaries. Further experimental measurements of the material parameters will provide better input to the theoretical modeling. The model can be further enhanced by considering the difference of Li

transport in the bulk and along/across the grain boundaries, and potential regulation of mechanical stresses on the rate of Li diffusion. While the current model can capture the salient features of mutual modulation between charge heterogeneity, bulk fracture, and surface passivation, the simplifications and uncertainties are also worth noting."

3. The main issue concern with this work is what appears to be a tenuous connection between the imaged particle and the simulation. The concern is because of the static imaging and the dynamic modeling and assigning the static observations to the dynamic simulations. Without knowing the beginning of life or initially discharged state of the particle, it is questionable whether conclusions should be drawn from images of a single particle.

Response:

We acknowledge the static nature of our experimental data, which is due to the practical constrains in the synchrotron experiments and we have thoroughly discussed this in our response to the first question from reviewer 2#. The scientific goal of this work is to elucidate the surface-to-bulk interplay that occurs on individual NMC particle. Although our experiment was not conducted inside a functioning battery cell, the particle level chemical and redox heterogeneity can persist even after long term relaxation [Adv. Mater. 28, 6631-6638 (2016)] and can be used as fossil evidence to reveal the correlation between the surface oxidation state and the bulk porosity, which is exactly what we observed in our data.

Regarding the beginning-of-life state of the particle, we have conducted a control experiment by imaging a pristine particle using the 3D XANES method (see the **Fig. R4a**). Here we plot the depth profiles of the Ni valence state over the four particles with different state of charge shown in **Fig. R5**. The depth profiles in **Fig. R5** clearly demonstrates a relative homogeneity in Ni's valence state throughout the pristine particle, while all the charged particles exhibit a depth dependent redox gradient.

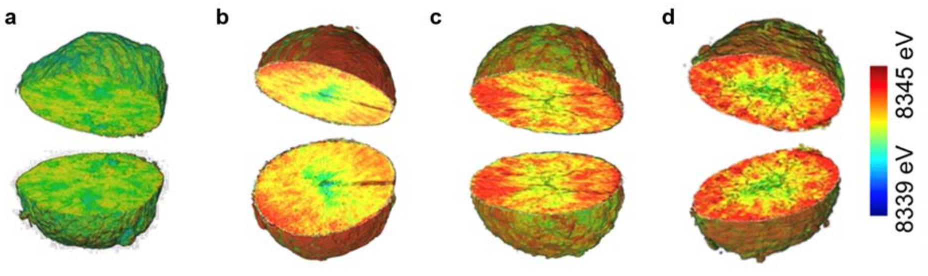

Fig. R4. 3D XANES mapping over the Ni K-edge for a few randomly selected NMC particles. The first particle (**a**) is at the pristine state and the rest of them are charged to different voltages (**b-d**). Regardless the degree of particle cracking, a depth dependent oxidation state distribution is clearly observed in the three charged particles, in which the surface regions are relatively more oxidized and the core regions are relatively more reduced. This observation suggests the overall outward Li diffusion, upon charging to high voltage. (unpublished data)

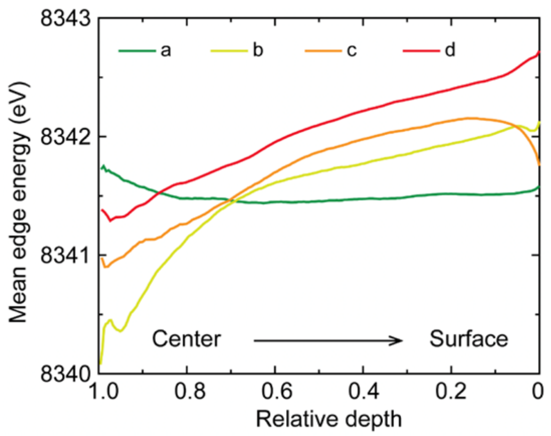

Fig. R5. The depth dependent Ni edge energy distribution over the four particles shown in **Fig. R4**. The green line (a) is the depth profile over the pristine particle, while the lines b, c, and d, are that of the particles at different state of charge. (unpublished data)

4. Was just the one particle imaged for each state, or is it just a representative example of an ensemble?

Response:

We thank the reviewer for this question. We again highlight the complexity and

challenge of the correlative imaging experiment, involving the utilization of multiple beamlines in a correlative manner. Our experiment is very time consuming, which makes it unpractical to scan many particles. The utilization of the soft x-ray nanoprobe also requires to extract single particle (it is not possible to utilize soft x-ray nanoprobe for measuring particles in an ensemble due to the limited penetration capability). We understand the concern regarding the representativeness and have conducted one more such measurement using another particle that is recovered from a different cell and the results are shown in **Fig. R6**. We clarify here that the data shown in **Fig. R6** has a lower spatial resolution due to the limitation in the beamtime available, but similar trend is observed in these two independent experiments.

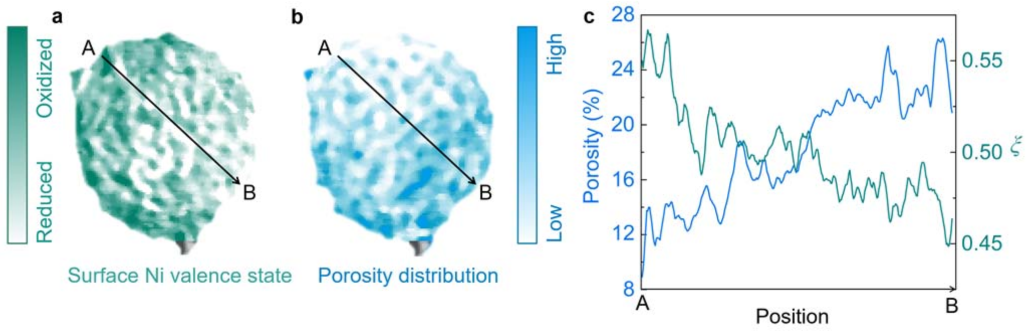

Fig. R6. The correlation between the local surface reconstruction and the bulk porosity. a, The surface (~5 nm depth) Ni valence state distribution. **b**, The bulk porosity map. **c**, The line profile from points A to B as illustrated in (**a**) and (**b**).

5. Is it possible that Pt welding for the sample mount of the particle impacted the observed valence state? The difference in the surface valence seems to vary with distance from the weld.

Response:

We do not anticipate any chemical reactions between the Pt and the particle. It is possible that the particle surface that is covered by Pt can have a different TEY response to the incident x-rays. It is, however, demonstrated in the hard x-ray tomographic data and the SEM image (see **Fig. R7** below and Supplementary **Fig. 5**)

that the Pt only exist near the tip of the W needle. The correlation analysis between soft x-ray nanoprobe data and hard x-ray tomographic data is executed over the entire particle surface, which is not severely affected by the localized Pt deposition. We therefore rule out the artifact caused by Pt welding.

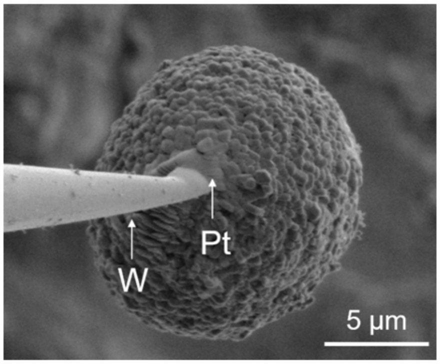

Fig. R7. SEM image of single NMC811 secondary particle mounted on the W needle with Pt welding.

6. Line 289: Where does the assumption of 1% Li diffusivity in the passivation layer come from? Is there data to support this, or is it just a modeling assumption?

Response:

This is an assumption of the modeling. While the literature [Adv. Energy Mater. 4, 1300787 (2014)] suggests that Li diffusion through the rock salt phase of the surface transformation layer is considerably slower than in NMC, we do not find a quantitative measurement of Li diffusivity in the surface passivation layer. This is perhaps because of the chemical complexity of the surface layer and its thin thickness. We have noted this assumption in the revised manuscript. Please see the response to comment 2.

7. The dynamic fracture processes the model predicts is not evident in the imaging.

Response:

We appreciate this comment. We have clarified and acknowledged the static nature of our experimental data. We have also thoroughly discussed the experimental

challenges and the practical limitations of our approach in the response to the comments listed above. On the other hand, we investigate the dynamic aspect of the complicated chemo-mechanical interplay in the system in our modeling effort. We have submitted the animation of the dynamic fracture process as a Supplementary file. The time evolution of the intergranular fracture along the grain boundaries with considering the liquid electrolyte penetration is visualized in the animation.

Revision:

We have added the following discussions in the 3rd paragraph of "Theoretical insight into the surface-to-bulk mutual modulation" section.

"In addition, as shown in Supplementary Movie 1, the time evolution of the intergranular fracture along the grain boundaries with considering the liquid electrolyte penetration is visualized in the animation."

8. Various typos, misspellings, and grammatical errors need to be corrected. The manuscript needs some careful editing: Line 29 "severer", Line 37 "LIB" should be "LIBs".

Response:

We thank the reviewer for pointing out this mistake. We have carefully gone through the manuscript and revised the errors as suggested.

General response and remarks:

We would like to take this chance to thank all the unnamed referees for the invaluable advice and suggestions, which help and guide us to rethink and polish our work greatly. We have tried our best to answer the questions from the reviewers. We believe that our revision has significantly improved the quality of our paper and we look forward to hearing your opinions on our submission.

Yijin Liu, Kejie Zhao, Jun-Sik Lee and Jieshan Qiu

On behalf of all co-authors

REVIEWERS' COMMENTS:

Reviewer #1 (Remarks to the Author):

The authors have addressed my comments. I recommend publication in the current form.

Reviewer #2 (Remarks to the Author):

The authors have done a detailed job of addressing the concerns in the first submission. In many cases, the concerns stem from overarching limitations in the capabilities of existing techniques at this time (e.g., temporal resolution) and outside the control of the research. The challenges in experiments are understandable.
